# Vitamins E and C supplementation reduces hepatic lipid peroxidation and steatosis in neonatal Yucatan piglets receiving prolonged parenteral nutrition

**DOI:** 10.1371/journal.pone.0361191

**Published:** 2026-11-02

**Authors:** Thilini Kumarasinghe, Amina S. Khaled, Robert F. Bertolo, Janet A. Brunton

**Affiliations:** Department of Biochemistry, Memorial University of Newfoundland, St. John’s, NL, Canada; Ankara University: Ankara Universitesi, TÜRKIYE

## Abstract

Parenteral nutrition (PN) is essential in neonatal care for many infants born prematurely or with impaired gastrointestinal function. However, prolonged PN use is associated with complications such as PN-associated liver disease and intestinal atrophy, potentially related to oxidative stress. Commercial PN lipid emulsions with fish oil are used to counteract PN-associated liver damage but may also contribute to oxidative stress due to their high content of long-chain polyunsaturated fatty acids. Using neonatal Yucatan miniature piglets as an established animal model for investigating PN-associated complications in neonates, this study investigated whether co-supplementation of vitamins E and C could reduce oxidative stress-related injury in the liver and small intestine during PN. The objective of this study was to determine whether supplementing a mixed-lipid emulsion (SMOFlipid®) with vitamins E and C would attenuate hepatic oxidative damage and enhance intestinal perfusion and morphology in neonatal piglets. Neonatal Yucatan piglets were surgically fitted with intravenous catheters and an indwelling blood flow probe around the superior mesenteric artery (SMA). Piglets received either standard PN (Control; n = 10) or PN supplemented with vitamins E (10 mg/kg/d) and C (100 mg/kg/d) (Vit E&C, n = 10) for ten days. Vitamin supplementation was associated with lower hepatic lipid accumulation and lipid peroxidation (P < 0.05 for both), compared with the control group. Plasma and liver vitamin E concentrations were markedly higher in the Vit E&C group (P < 0.001 and P < 0.01, respectively). No differences were determined between groups in liver or plasma triglyceride and cholesterol levels, SMA blood flow, intestinal villus height, crypt depth, or overall intestinal morphology. Hepatic and intestinal protein synthesis rates and in vivo bile acid clearance were also not significantly different between groups. Co-supplementation of PN with vitamins E and C was associated with lower hepatic oxidative stress and steatosis in neonatal piglets. Although no improvements were measured in intestinal structure or perfusion, these findings support the potential of antioxidant-enriched PN formulations to mitigate PN-associated liver complications.

## Introduction

One of the most prominent challenges experienced by neonatal clinicians is providing optimal nutrition for premature and sick infants, particularly when enteral feeding is contraindicated. In such cases, parenteral nutrition (PN) becomes essential. However, PN remains a poorly defined intervention in neonatal care and is associated with serious complications when used long term, including gut mucosal atrophy and adverse liver outcomes. These complications arise from bypassing first-pass splanchnic metabolism, leading to a rapid suppression of intestinal blood flow and subsequent gut mucosal atrophy, along with oxidative stress and inflammation [1,2]. Gut atrophy impairs metabolic functions, increases the risk for necrotizing enterocolitis, and delays the transition to enteral feeding [1,3,4]. Liver damage that progresses to PN-associated liver disease (PNALD) is characterized by cholestasis, hepatic steatosis, and fibrosis with elevated conjugated bilirubin and liver enzymes as clinical markers [5,6].

To investigate these complications under controlled experimental conditions, this study used neonatal Yucatan miniature piglets, an established animal model for investigating PN-associated complications in newborns. Neonatal piglets share many physiological and gastrointestinal developmental characteristics with human infants, particularly preterm infants, making them a relevant translational model for neonatal nutrition research [7,8].

Oxidative stress, a key driver of PNALD, arises from an imbalance between reactive oxygen species (ROS) and antioxidant defences [9]. ROS damage proteins, lipids, and DNA, triggering inflammation and lipid peroxidation [10,11]. Premature infants are particularly vulnerable to oxidative damage due to underdeveloped antioxidant systems and stressors such as hypoxia and hypothermia [12,13]. Additionally, PN solutions can generate oxidants through interactions among the elemental nutrients, especially in the presence of light or oxygen, producing cytotoxic, vasoconstrictive compounds that further impair gut perfusion and promote atrophy [14,15]. Vitamins E and C are potent antioxidants that may provide complementary antioxidant effects, including protection against lipid peroxidation. Synergistic interactions between vitamins E and C have been demonstrated in in vitro systems [16], while evidence in vivo remains mixed. For example, vitamin C has been shown to spare vitamin E and increase tissue α-tocopherol concentrations in a rodent model unable to synthesize vitamin C [17], whereas a randomized factorial trial in human adults found no additional benefit of combined vitamins E and C supplementation on lipid peroxidation compared with either vitamin alone [18]. Despite mixed findings, their combined use is common among athletes to enhance exercise performance [19]. Given their complementary antioxidant functions, investigating the effects of combined vitamin E and C supplementation in the PN context is important, as oxidative stress remains a central concern.

New-generation fish oil-based lipid emulsions are manufactured with added vitamin E to prevent peroxidation of longer-chain n-3 polyunsaturated fatty acids (PUFAs), but their impact on the antioxidant status of the patient is not fully understood. For instance, a piglet study showed improvements in PNALD markers with added vitamin E, however plasma vitamin E concentration remained below recommended thresholds for PN-fed infants, suggesting that the total vitamin E provided by PN may be too low [6]. Moreover, vitamin E degrades by photooxidation in PN solutions [9]. As an antioxidant, vitamin C scavenges reactive oxygen species and importantly can regenerate oxidized vitamin E both in vivo and in vitro, potentially enhancing systemic antioxidant protection and improving the oxidative stability of PN formulations [20]. It should be noted that vitamin C can be synthesized in pigs, so the addition of vitamin C to PN is used here to evaluate its role in enhancing the effectiveness of vitamin E.

We hypothesized that supplementing a fish oil-containing lipid emulsion with vitamins E and C during prolonged PN in neonatal piglets would reduce oxidative stress and lipid peroxidation, attenuate liver injury associated with PNALD, reduce the suppression of blood flow to the intestine, and reduce gut atrophy. The specific objectives of this study were to investigate: 1) the effects of a lipid emulsion supplemented with vitamin E and vitamin C on liver pathology, markers of oxidative stress, liver protein synthesis, and liver and plasma concentrations of α-tocopherol; 2) the effects of a lipid emulsion supplemented with vitamin E and vitamin C on superior mesenteric artery blood flow, gut morphology, markers of oxidative stress, and intestinal protein synthesis.

## Materials and methods

### Ethics statement

All animal procedures were approved by the Memorial University of Newfoundland Animal Care Committee (protocol number: 19−02 JB) and conducted in accordance with the guidelines of the Canadian Council on Animal Care. Animals received continuous veterinary oversight, and all surgical procedures were performed by trained research personnel, with a veterinary technician present and under the supervision of a clinical veterinarian. Animals were monitored at least twice daily for general health, behavior, catheter integrity, and signs of pain or distress. Following surgical catheterization, animals received parenteral nutrition for a total study duration of 10 days, after which a planned terminal necropsy was performed. Humane endpoints were predefined, with a plan to humanely euthanize the animals prior to the planned study endpoint if severe or unrelieved pain, infection, or clinical deterioration occurred. A total of twenty-one neonatal Yucatan miniature piglets entered the study over the course of the experiment. One animal allocated to the Vit E&C group was humanely euthanized on study day six due to clinical deterioration and was excluded from all analyses. An additional piglet was subsequently enrolled to maintain the planned final sample size. Twenty piglets completed the study and were included in the final analyses.

### Piglets, surgical procedures and daily care

Yucatan piglets (10–14 days of age; initial body weight 2.13 ± 0.26 kg) were obtained from a breeding herd at Memorial University of Newfoundland (St. John’s, Canada). Randomization of piglets to the two treatments was blocked by littermate pairs (n = 10 per treatment). Upon arrival, piglets underwent surgery under general anesthesia for implantation of two venous catheters, as previously described [21]. Briefly, a femoral venous catheter was advanced into the inferior vena cava to facilitate repeated blood sampling and administration of medications, while a jugular venous catheter was advanced into the superior vena cava for continuous parenteral nutrition infusion. An ultrasonic perivascular blood flow probe (4–6 mm; Transonic Systems, Ithaca, NY) was also surgically implanted around the superior mesenteric artery (SMA) to allow for chronic measurement of intestinal blood flow. The SMA was isolated below the left kidney adjacent to the abdominal aorta, and the probe was secured around the vessel. The probe cable was anchored to the abdominal musculature, tunnelled subcutaneously, and exteriorized dorsally with the intravenous catheters. Blood flow was assessed before incision closure to confirm probe function. Postoperative care included administration of prophylactic antibiotics (trimethoprim and sulfadoxine; Trimidox, Vetoquinol, Lavaltrie, QC, Canada) and analgesia (buprenorphine; Bupaq; CDMV, Saint-Hyacinthe, QC, Canada). Following surgery, piglets were housed individually in metabolic cages equipped with a swivel and tether system (Lomir Biomedical) that allowed free movement while delivering continuous intravenous infusions. Room temperature was maintained between 28°C and 32°C using supplementary heat lamps, and the room was on a 12-hour light/dark cycle.

All piglets received a complete PN solution (described below). On the day of surgery (day 0), PN was initiated at 6 mL/kg/h corresponding to 50% of the total nutrient requirements. The infusion rate was increased to 75% on the morning of day 1, and to the final rate of 12 mL/kg/h on the evening of day 1. From day 2 onward, piglets were weighed daily, and infusion rates were adjusted accordingly. Buprenorphine was administered from day 1 to day 3 and Trimidox from day 0 to day 4.

For reference purposes, blood samples were collected from a separate group of age-matched, sow-reared piglets (n = 6) to measure baseline plasma vitamin E concentrations. These reference animals did not undergo surgery or receive parenteral nutrition.

### Diets

All piglets received a PN diet formulated specifically for neonatal piglets, based on the neonatal piglet PN formulation described by Wykes et al. [22], and modified for PN-fed Yucatan miniature piglets according to Brunton et al. [23]. The elemental diets (S1 Table) were prepared in our laboratory under aseptic conditions following the PN preparation procedure described by Dodge et al. [21]. Briefly, crystalline amino acids were dissolved in water under a blanket of nitrogen, followed by the addition of dextrose and minerals. The solution was then filter sterilized, transferred into sterile intravenous infusion bags, and stored in refrigeration until use.

Both the treatment and control diets provided 0.9 MJ of metabolizable energy per kilogram of body weight per day, with dextrose (21.6 g/kg/d), lipids (9.3 g/kg/d as 20% SMOFlipid®, Fresenius Kabi), and proteins (13.1 g/kg/d as free amino acids). All diets were supplemented with vitamins and macro minerals at levels exceeding 100% of the estimated requirements for neonatal piglets according to NRC guidelines (1998). Immediately before infusion, multivitamins, iron dextran (Bimeda-MTC Animal Health), and trace elements were added aseptically to each diet bag. Diets were continuously infused (24 h/d) via pressure-sensitive peristaltic pumps. The vitamin supplemented group (Vit E&C; n = 10) received the same PN diet as the Control group, but with additional vitamin E (α-tocopherol) and vitamin C (ascorbic acid). Vitamin E was supplemented to provide a total dose of 10 mg/kg/d (compared to 3.4 mg/kg/d in Control), a concentration previously shown to attenuate PNALD progression in piglet models, while maintaining plasma levels below the American Academy of Pediatrics’ upper recommended limit for parenterally fed preterm infants [6]. Vitamin C was added to provide a total dose of 100 mg/kg/d (compared to 15.3 mg/kg/d in Control). While the ideal parenteral nutrition ratio of vitamins E to C has not been established, previous oxidative stress studies in other models have demonstrated that co-administration enhances vitamin E’s antioxidant effects [20,24]. A 1:10 ratio (vitamin E: vitamin C) has been applied clinically in acute oxidative stress [9].

### SMA blood flow reading and blood sampling

The implanted SMA flow probe was connected to a Transonic T403 flowmeter (Transonic Systems) via an extension cord. Piglets remained unrestrained in their cages during the measurement periods. Blood flow rate was recorded for 10-minute intervals every 12 h starting from day 1 post-surgery. SMA blood flow values were first normalized to body weight (mL/min/kg) and then expressed as a percentage relative to the baseline flow measured during surgery (baseline = 100%). Thus, data are reported as SMA blood flow (% baseline).

Blood samples (2 mL) were collected every other day beginning on day 2. Samples were drawn into EDTA-treated tubes (BD Biosciences, Mississauga, Ontario, Canada) and processed for plasma separation.

### Stable isotope infusion and necropsy procedure

Stable isotopic tracer approaches were used to evaluate the effects of treatment on bile acid clearance and tissue specific protein synthesis rates. On day 8, piglets received a bolus intravenous injection of 24-^13^C chenodeoxycholic acid (24-^13^C-CDCA, 99%, Cambridge Isotope Laboratories, Tewksbury, Massachusetts, USA) at a dose of 25 mg/kg body weight. The tracer was prepared at a concentration of 100 mg/mL in ethanol. Blood samples were collected before the infusion and then every 3 h for 12 h, followed by additional samples at 18, 24, 36, and 48 h. On day 10, a flooding bolus dose of unlabeled L-phenylalanine (1.5 mmol/kg body weight; Sigma-Aldrich) and D_8_-labeled L-phenylalanine (0.15 mmol/kg body weight, 98% enrichment; Cambridge Isotope Laboratories, Inc.) was administered over 5 min. After 30 min, necropsy was initiated. The piglets were moved to the surgical suite and placed under general anesthesia. The abdominal cavity was opened, and the length and weight of the entire small intestine were measured. A 60-cm section of the proximal jejunum was rinsed with ice-cold saline, opened longitudinally, placed on a glass plate on ice, and the mucosa was scraped using a glass slide. The mucosal scrapings were weighed and flash-frozen in liquid nitrogen. Additionally, a 1 cm segment was placed in neutral buffered 10% formalin for histological examination. Organs were excised, weighed, and tissue samples were immediately frozen using a freeze clamp and liquid nitrogen, then stored at −80°C for further analyses. The correct placement of the SMA blood flow probe was verified during necropsy. Piglets were euthanized by exsanguination following organ removal.

## Analysis procedure

### Histology

Jejunal tissue samples were fixed in 10% neutral buffered formalin and submitted to the Histology Laboratory, Faculty of Medicine, Memorial University (St. John’s, NL, Canada) for processing, slide preparation and haematoxylin and eosin (H&E) staining. Villus height was measured from the tip of the villus to the villus-crypt junction, whereas crypt depth was measured from the villus-crypt junction to the base of the crypt [25]. Ten well-oriented villus-crypt units were measured per sample by the same individual blinded to treatment allocation using a Zeiss Axiostar microscope (Carl Zeiss Canada Inc., Toronto, ON, Canada). Images were captured and analyzed using a ToupView software version 4.11 (ToupTek, Hangzhou, Zhejiang, China).

### Antioxidant capacity and lipid peroxidation analyses

Ferric reducing antioxidant power (FRAP) assay was done to measure the antioxidant capacity of the liver. Liver samples were prepared using a previously described extraction procedure [26]. Briefly, 100 mg of frozen liver tissue was homogenized in 1.0 ml of 1.15% KCl (10% w/v), centrifuged at 277 g for 10 min at 4 °C, and the resulting supernatant was diluted 1:5 with deionized water. Antioxidant capacity was then determined using the diluted supernatant using a commercial FRAP assay kit (ab234626; Abcam, Cambridge, UK) according to the manufacturer’s instructions.

The extent of lipid peroxidation in liver and plasma was assessed using the thiobarbituric acid reactive substances (TBARS) assay, which quantifies malondialdehyde (MDA), a marker of oxidative stress [27]. Briefly, 100 mg of tissue was homogenized in 1 mL phosphate-buffered saline (PBS) and centrifuged at 3000 *g* for 10 min. The supernatant (100 µL) was mixed with 100 µL of 0.9% NaCl, 200 µL of 8% trichloroacetic acid (TCA), 100 µL of 2.5 mM butylated hydroxy toluene (BHT), 150 µL of 2.9 mM thiobarbituric acid (TBA), and 60 µL of 8.1% sodium dodecyl sulfate (SDS). Samples were vortexed briefly, heated at 95 °C for 60 min, cooled on ice for 10 min, and extracted with 750 µL n-butanol, followed by centrifugation at 1400 g for 20 min at 4 °C. Absorbance was measured at 532 nm using a microplate reader within 30 min. A standard curve was generated using serial dilutions of tetramethoxypropane (TMP), and sample MDA concentrations were calculated from the standard curve and expressed as MDA equivalents.

### Lipids and bile acid analyses

Lipids were isolated from liver tissue using the chloroform: methanol (2:1, vol/vol) extraction method [28]. The extracted lipids were then dried under a stream of nitrogen gas and weighed to measure the total lipid content. These dried lipids were subsequently dissolved in isopropanol and analyzed for hepatic cholesterol and triglyceride (TG) levels using enzymatic colorimetric assay kits (Sekisui Diagnostics, Charlottetown, Prince Edward Island, Canada; catalog numbers 236−60 for cholesterol and 234−60 for TG). Plasma samples were analyzed using the same commercial kits. For assay calibration, a TG standard (26 mg/dL; Sigma-Aldrich®) and a cholesterol standard (100 mg/dL; FUJIFILM Wako Pure Chemical Corporation) were used to generate standard dilution curves, and absorbance readings from the samples and standards were obtained using a microplate reader (Synergy™ Mx Fluorescence/UV-Vis, BioTek Instruments). In addition, bile from the same piglets was analyzed for phosphatidylcholine, cholesterol, and bile acids, while liver samples were analyzed for phosphatidylcholine [29,30] (see S1 File).

### Liver dry matter

Liver dry matter was assessed by weighing approximately 1 g of frozen tissue sample, drying at 100 ^o^C for 24 h, and reweighing. Liver dry matter was expressed as a percentage of the initial wet tissue weight.

### Tissue-specific rates of protein synthesis

Fractional rate of protein synthesis (FSR) was determined in liver and jejunal mucosa based on the modified method adapted from Lamarre et al. [31]. The isotopic enrichment of L-phenylalanine (D_8_) in free and protein-bound tissue fractions was assessed using pentafluorobenzyl bromide (PFBBr) derivatives and analyzed by gas chromatography-mass spectrometry (GC-MS; 8890 GC coupled with a 5977 MSD/FID, Agilent Technologies, Palo Alto, CA).

The percentage molar enrichment (mol%) was calculated for both free (IE_free_) and protein-bound (IE_bound_) phenylalanine by analyzing isotopic enrichment of L-phenylalanine (D_8_) in the PCA-insoluble (protein-bound) and PCA-soluble (tissue-free) phenylalanine pools:


$$\begin{array}{c} FSR\left(\%\right)=\left(\mathrm{IEbound}/\mathrm{IEfree}\right)\cdot \left(1440/t\right)\cdot 100 \end{array}$$

The total number of minutes in a day is 1440, and (t) represents the duration in minutes that the sample was labelled, determined by the time elapsed until the tissue was frozen following the isotope flooding dose.

### Vitamin E analysis

Vitamin E content in plasma, liver and jejunal mucosa was measured using Acquity ultra-performance liquid chromatography (UPLC) system (Waters Corporation, Mississauga, ON, Canada). Plasma samples were prepared through a liquid-liquid extraction method as reported previously [32]. Separation was performed using a Zorbax Eclipse XDB-C18 column (150 mm x 4.6 mm x i.d., 5 µm particle size; Agilent Technologies, Palo Alto, CA). Methanol served as mobile phase A, while acetonitrile was mobile phase B. The run was completed in 20 min under isocratic conditions at 30% A and 70% B at a flow rate of 1 mL/min. Retinol acetate, used as the internal standard, was detected at an excitation wavelength of 325 nm and an emission wavelength of 470 nm, whereas α-tocopherol was detected at an excitation wavelength of 295 nm and an emission wavelength of 330 nm. Liver and intestinal mucosa samples were prepared according to a previously described method with slight modifications [33]. Briefly, tissue samples weighing 100 mg were homogenized with 1 mL of water. Then, 160 µL of an internal standard solution (retinol acetate at 25 μg/mL in ethanol) and 400 µL of ethanol containing 0.04% BHT were added. The mixture was vortex-mixed for 10 s. Next, 800 µL of hexane with 0.04% BHT was added, and the mixture was vortex-mixed for 3 min. This was followed by centrifugation at 3500 × g for 10 min at 10 °C. From the resulting layers, 700 µL of the hexane layer was carefully transferred to a borosilicate glass tube. The hexane was evaporated using nitrogen gas, and the remaining pellet was resuspended in 200 µL of methanol. This solution was vortex-mixed for 30 s and transferred into amber vials. Then 20 µL of tissue extract was injected into the UPLC system for analysis, using the same chromatographic conditions as those applied to plasma samples.

### Chenodeoxycholic acid analysis

Plasma bile acids were extracted using a solid-phase extraction method, and isotopic enrichments in plasma were subsequently assessed using liquid chromatography-tandem mass spectrometry (LC-MS TOF; 1260 LC- 6230 MS TOF; Agilent Technologies) following a previously established method [34]. Bile acid extraction from plasma was performed using solid-phase extraction (SPE) with 200 mg Bond Elut cartridges (Agilent Technologies, Mississauga, ON, CA). The cartridges were preconditioned sequentially with 2 mL methanol, 2 mL LC-MS grade water, and 2 mL of 100 mmol/L ammonium carbonate buffer (pH 9.3). Plasma samples were mixed with an appropriate amount of internal standard, ^2^H₅-chenodeoxycholic acid (2,2,3,4,4-D5 CDCA,98%, Cambridge Isotope Laboratories, Tewksbury, Massachusetts, USA), 100 µL of 100 mmol/L ammonium carbonate buffer (pH 9.3), and 500 µL LC-MS grade water before being applied to the SPE columns. Following gravity flow, the columns were washed with 2 mL LC-MS grade water and dried under vacuum. Bile acids were eluted using 3 mL methanol, evaporated to dryness, and reconstituted in 60 µL LC-MS grade methanol and 30 µL of 10 mM ammonium acetate buffer (pH 6.5) prior to LC-MS TOF analysis. Chromatographic separation was achieved using a 150 mm × 2.1 mm x 5 µm Zorbax RX-SIL column (Agilent Technologies, Mississauga, ON, CA). The mobile phases were methanol and 10 mM ammonium acetate buffer (pH 6.5), applied in a gradient. Electrospray ionization was performed in negative mode.

### Statistical analysis

The planned sample size of eight piglets per treatment group was determined based on variance estimates from previous SMA blood flow measurements in neonatal piglets, with a significance level of 0.05 and 80% power to detect a 15% difference in SMA blood flow [35]. However, the planned sample size was increased to 10 piglets per treatment group to increase statistical power for the assessment of other study outcomes. All available data from the 20 piglets completing the study were included in the analyses unless outcome-specific measurements were unavailable because of technical or procedural limitations. For SMA blood flow analysis, four piglets (two per treatment group) had absent or incomplete flow-probe recordings, resulting in n = 8 per group. For the ^13^C-CDCA clearance analysis, one Control piglet was not assessed because of a nonfunctional femoral catheter, resulting in n = 9 Control and n = 10 Vit E&C. Outcome-specific sample sizes are reported in the corresponding figure legends.

Differences in treatment means for discrete variables were analyzed using a t-test. For SMA blood flow data, a two-way repeated measures ANOVA (treatment × day) was initially performed. As no significant effect of treatment or treatment × day interaction was observed, data were pooled, and the main effect of time was then assessed. Post hoc comparisons were conducted using Dunnett’s test, comparing each day to baseline (day 0). Simple linear regression analyses were performed to evaluate relationships between hepatic lipids (TG, cholesterol) and total liver lipid content within each group. Regression slopes and intercepts were compared between treatment groups. In addition, regression of hepatic antioxidant capacity (FRAP) against initial body weight was performed using pooled data from all piglets. The kinetics of ^13^C-CDCA clearance were modeled using an exponential decay model. Statistical software used for data analysis was GraphPad Prism 10 (Graph Pad Software, San Diego, CA, USA). The alpha level was set at 0.05. All results were reported as mean ± standard deviation (SD), except for SMA blood flow data, which are presented as mean ± standard error of the mean (SEM).

Investigators were aware of treatment allocation during the conduct of the experiment because treatment-specific PN formulations were administered to each piglet. Biological samples were identified by study code rather than treatment group, and personnel performing these assays were unaware of treatment allocation.

## Results

### Growth and organ development

The body weight of all piglets increased over the course of the experiment and was not affected by the treatment (Table 1). Similarly, there were no differences in organ weights or liver dry matter between the treatment groups (Table 1).

**Table 1:** Growth rate and organ weights at necropsy.

Variable	Control	Vit E&C	P value
Initial body weight (kg)	2.16 ± 0.31	2.09 ± 0.21	0.58
Final weight (kg)	3.30 ± 0.43	3.24 ± 0.36	0.74
Growth rate (g/kg body weight/d)	52 ± 9	54 ± 8	0.69
SI weight (g/kg body weight)	17 ± 1	19 ± 2	0.25
SI length (cm/kg body weight)	204 ± 24	209 ± 20	0.58
Liver wet weight (g/kg body weight)	39 ± 5	36 ± 1	0.16
Liver dry matter (%)	27.35 ± 0.84	27.53 ± 2.09	0.80
Kidney wet weight (g/kg body weight)	4.31 ± 0.70	4.25 ± 0.66	0.83
Brain wet weight (g/kg body weight)	11.18 ± 0.58	11.03 ± 1.20	0.74

Values are means ± SD; n = 10 for each treatment group. P values were determined by t-test.

### Jejunal histology

Supplementation with vitamins E and C did not appear to affect several morphological parameters of the small intestine. Specifically, there were no differences in the circumference of the jejunum, the weight of the jejunal mucosa, the height of the jejunal villi (VH), the depth of the crypts (CD), or the villus height to crypt depth ratio (VH/CD) (Table 2).

**Table 2:** Intestinal morphology at necropsy.

Parameter	Control	Vit E & C	P value
Jejunal circumference (cm/kg)	0.62 ± 0.17	0.62 ± 0.14	0.99
Jejunal mucosal weight (mg/cm)	48 ± 17	51 ± 13	0.70
Jejunal villus height (µm)	222 ± 74	219 ± 34	0.29
Jejunal crypt depth (µm)	109 ± 31	113 ± 11	0.47
Jejunal VH/CD ratio	2.04 ± 0.29	1.95 ± 0.37	0.56

Values are means ± SD; n = 10 for each treatment group. P values were determined by t-test

### Superior mesenteric artery blood flow

The study explored the impact of antioxidant vitamin supplementation on blood flow in the superior mesenteric artery during parenteral feeding, with the objective of understanding their influence on vascular dynamics during PN. Contrary to our hypothesis, supplemental vitamins E and C did not attenuate the PN-induced decline in blood flow throughout the study period (Fig 1). In both groups, blood flow to the gut progressively decreased over time with parenteral feeding. By day 3, there was a 35% reduction in blood flow, which further declined to 41% by day 4. Subsequently, blood flow remained relatively stable until day 10, regardless of the treatment administered.

**Fig 1:**
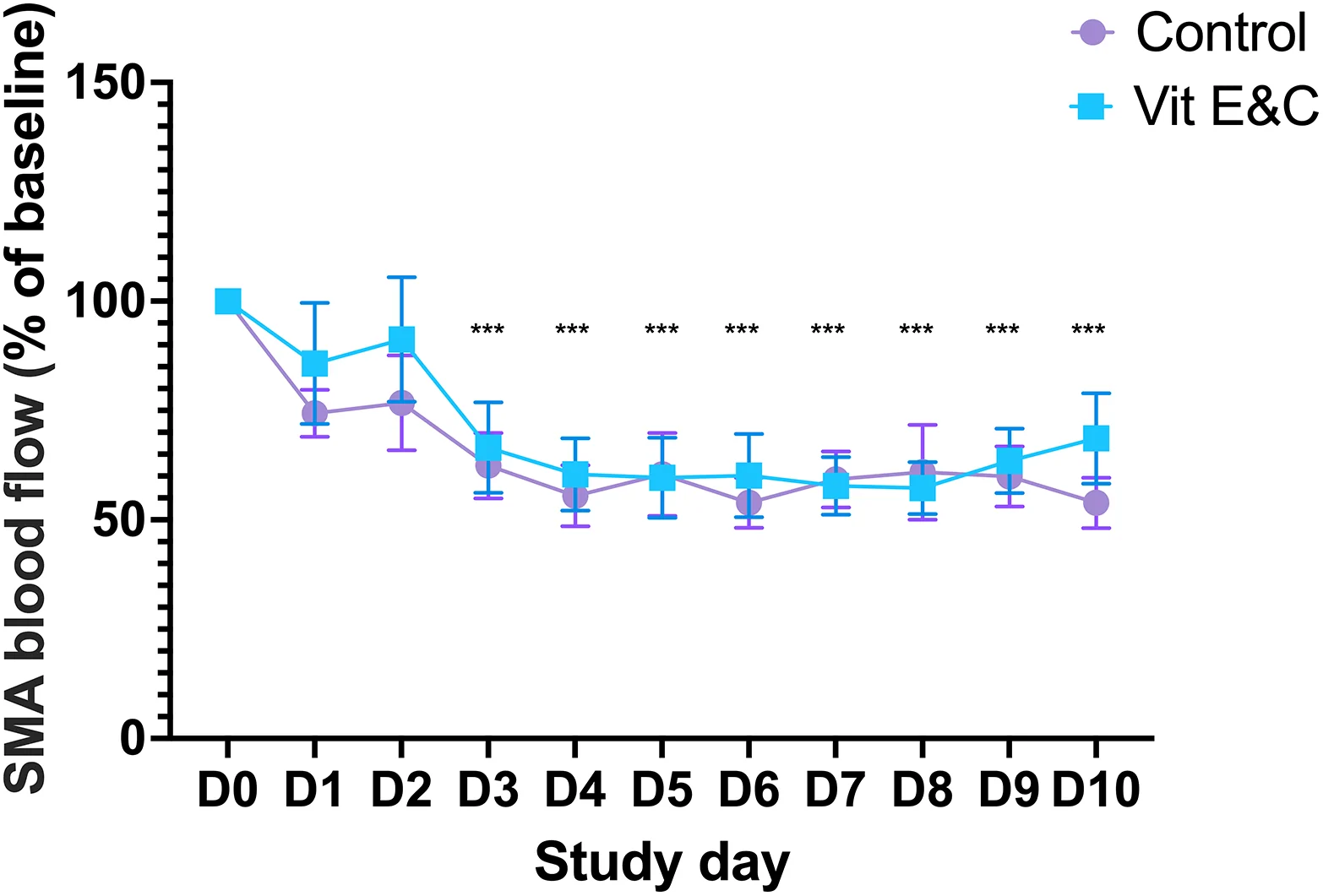
Daily SMA blood flow. SMA blood flow (% baseline) was analyzed by two-way repeated measures ANOVA (treatment group x day). No significant treatment effect was detected, but there was a significant main effect of time. Therefore Dunnett’s multiple comparisons test was performed on the pooled data to compare mean blood flow on each day to baseline (day 0). The asterisks above individual days indicate a significant difference from day 0 for the pooled data. Data are presented as mean ± SEM; n = 8 per group. ***P < 0.001.

### Antioxidant capacity and lipid peroxidation analyses

Hepatic lipid peroxidation (MDA equivalents) was higher in Control piglets compared with Vit E&C piglets (Table 3). Despite this, no significant differences were measured in MDA concentrations in jejunal tissue between the treatment groups. The antioxidant reducing power in the liver determined by the FRAP assay was not significantly different between treatments (P = 0.15). Body weight at study entry, used as a surrogate for developmental stage, was not significantly correlated with hepatic FRAP values (R^2^ = 0.06, P = 0.31; S1 Fig).

**Table 3:** Tissue and plasma lipid profiles, lipid peroxidation and antioxidant capacity.

Parameters	Control	Vit E & C	P value
Liver			
Total lipid (mg/g)	28.04 ± 8.46	20.88 ± 3.77	0.02*
Triglyceride (μmol/g)	3.65 ± 0.98	2.92 ± 1.10	0.37
Cholesterol (μmol/g)	4.03 ± 0.61	3.85 ± 0.63	0.53
FRAP equivalents (μmol/g)	366 ± 70	408 ± 55	0.15
TBARS; MDA equivalents (μmol/g)	0.87 ± 0.10	0.73 ± 0.15	0.03*
Plasma			
Total cholesterol (mmol/L)	1.56 ± 0.46	1.59 ± 0.40	0.87
Total triglycerides (mmol/L)	0.45 ± 0.33	0.34 ± 0.13	0.36
Small intestine mucosa (jejunum)			
TBARS; MDA equivalents (μmol/g)	0.37 ± 0.20	0.33 ± 0.13	0.64

Values are means ± SD; n = 10 for each treatment group. P values were determined by t-test. Significant differences between groups are indicated by * P < 0.05.

### Lipid and bile acid analyses

Vit E&C piglets had lower total lipid concentrations in the liver than Control piglets (Table 3). However, there were no differences in liver or plasma triglyceride and cholesterol levels (Table 3). To further explore lipid partitioning, we examined the linear relationships between hepatic TG and cholesterol with total liver fat. In Controls, liver cholesterol significantly predicted total liver fat, whereas liver TG was not a significant predictor (Fig. 2). In contrast, in Vit E&C piglets, hepatic TG significantly predicted total liver fat, while hepatic cholesterol did not (Fig 2). However, comparisons of the regression lines showed that the slopes did not differ significantly between treatment groups for either hepatic cholesterol (P = 0.33) or TG (P = 0.94).

**Fig 2:**
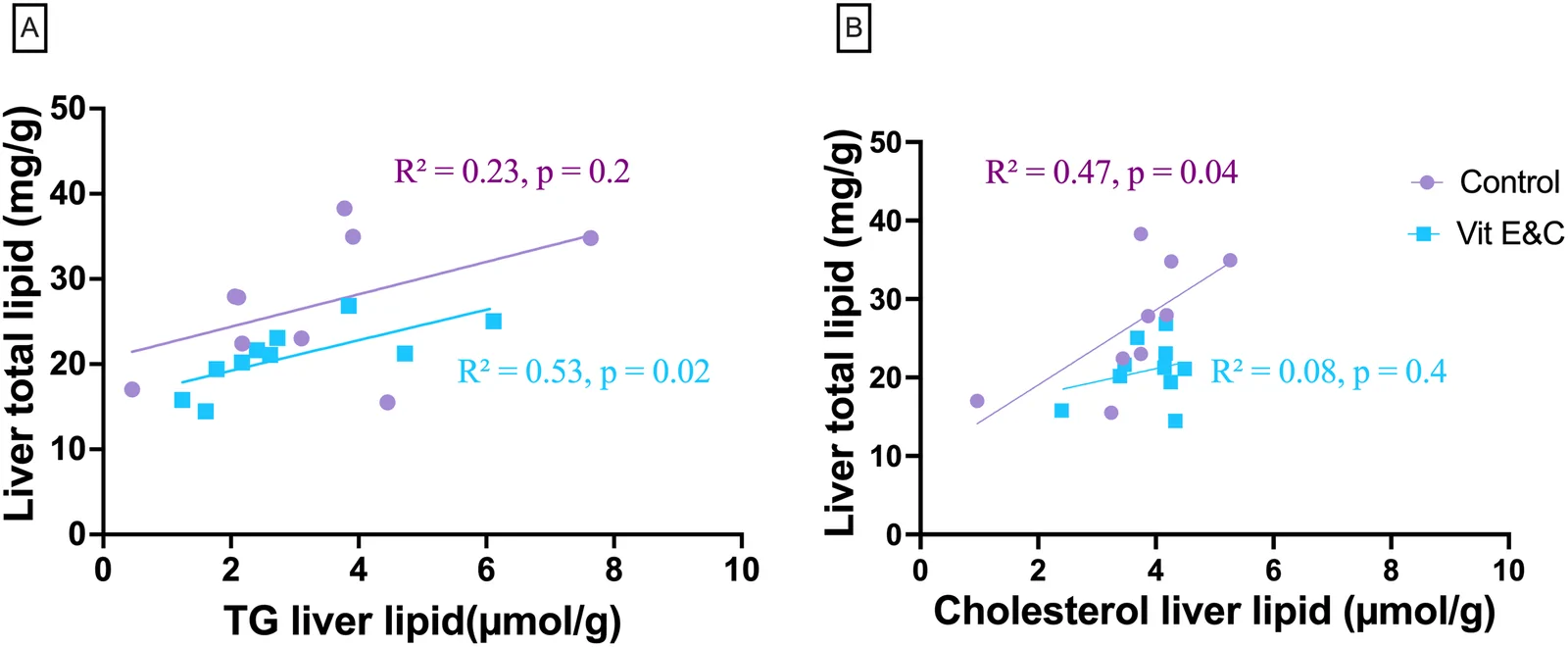
Relationships between hepatic TG and hepatic cholesterol with total liver lipid by treatment. Panel A shows hepatic TG, and panel B shows hepatic cholesterol, each plotted against total liver lipid. Control (purple) and vitamin E & C supplemented (blue) piglets are shown with separate linear regression fits.

Additional analyses of bile and liver samples also showed no significant differences in bile phosphatidylcholine, cholesterol, or bile acids, nor in liver phosphatidylcholine between groups (see S2 Table).

### Tissue-specific rates of protein synthesis

The fractional protein synthesis rate in the liver and intestine was not significantly different between treatment groups. In liver, the fractional protein synthesis rate was 80.3 ± 39.1%/d in the Vit E&C group versus 75.7 ± 37.0%/d in Control. Similarly, the small intestinal mucosa showed comparable rates between the supplemented (84.3 ± 34.1%/d) and the control (86.5 ± 46.0%/d) groups.

### Vitamin E analysis

The plasma concentration of vitamin E was approximately 3-fold higher in the Vit E&C group compared to the Control group (Fig 3), and 1.5-fold higher in liver tissue (Table 4). A group of sow-fed piglets (n = 6) that was included solely for reference purposes, had a mean plasma vitamin E concentration of 6.12 ± 0.71 μg/mL. Vitamin E concentrations in the intestinal mucosa were approximately ~3% of those in the liver, and were not affected by the treatment (Table 4).

**Fig 3:**
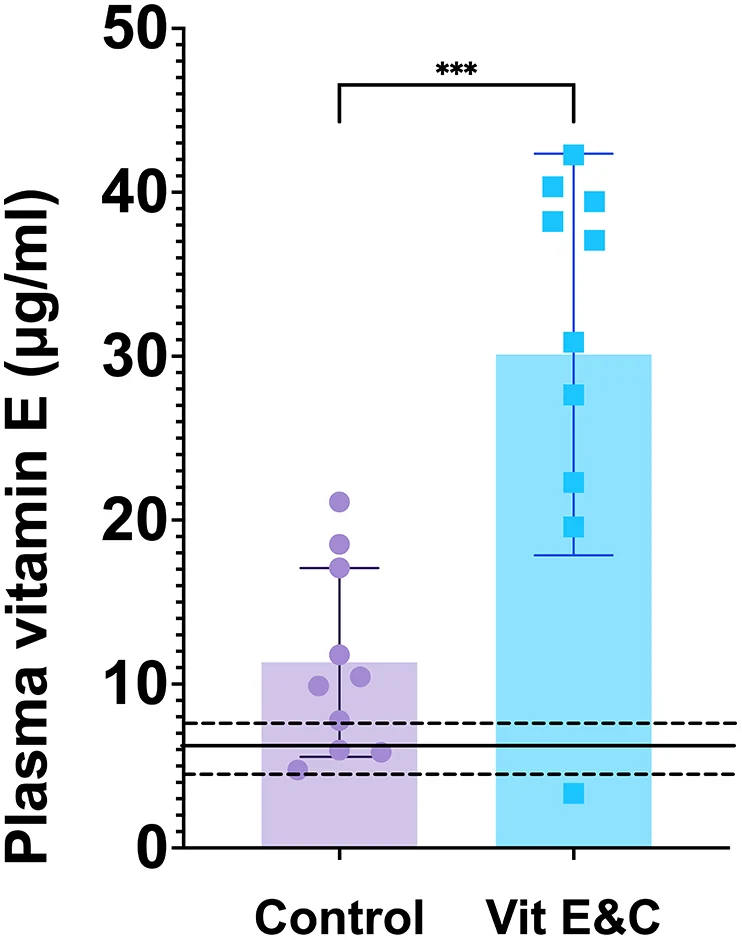
Plasma vitamin E concentration. Values are presented as means ± SD; n = 10 per group. P values were determined by unpaired t-test; ***P < 0.001. A group of sow-fed piglets (n = 6) was included for reference purposes, showing the group mean (solid line) ± two standard deviations (dashed lines).

**Table 4:** Vitamin E content in liver and jejunal mucosa.

	Control	Vit E & C	P value
Liver (µg/g)	120.3 ± 27.3	202.9 ± 64.6	0.002
Jejunal mucosa (µg/g)	4.83 ± 1.95	6.04 ± 2.53	0.30

Values are means ± SD; n = 10 for Control & Vit E & C group. P values were determined by t-test.

### Chenodeoxycholic acid analysis

Bile acid clearance between control and Vit E&C groups was not different (Fig 4).

**Fig 4:**
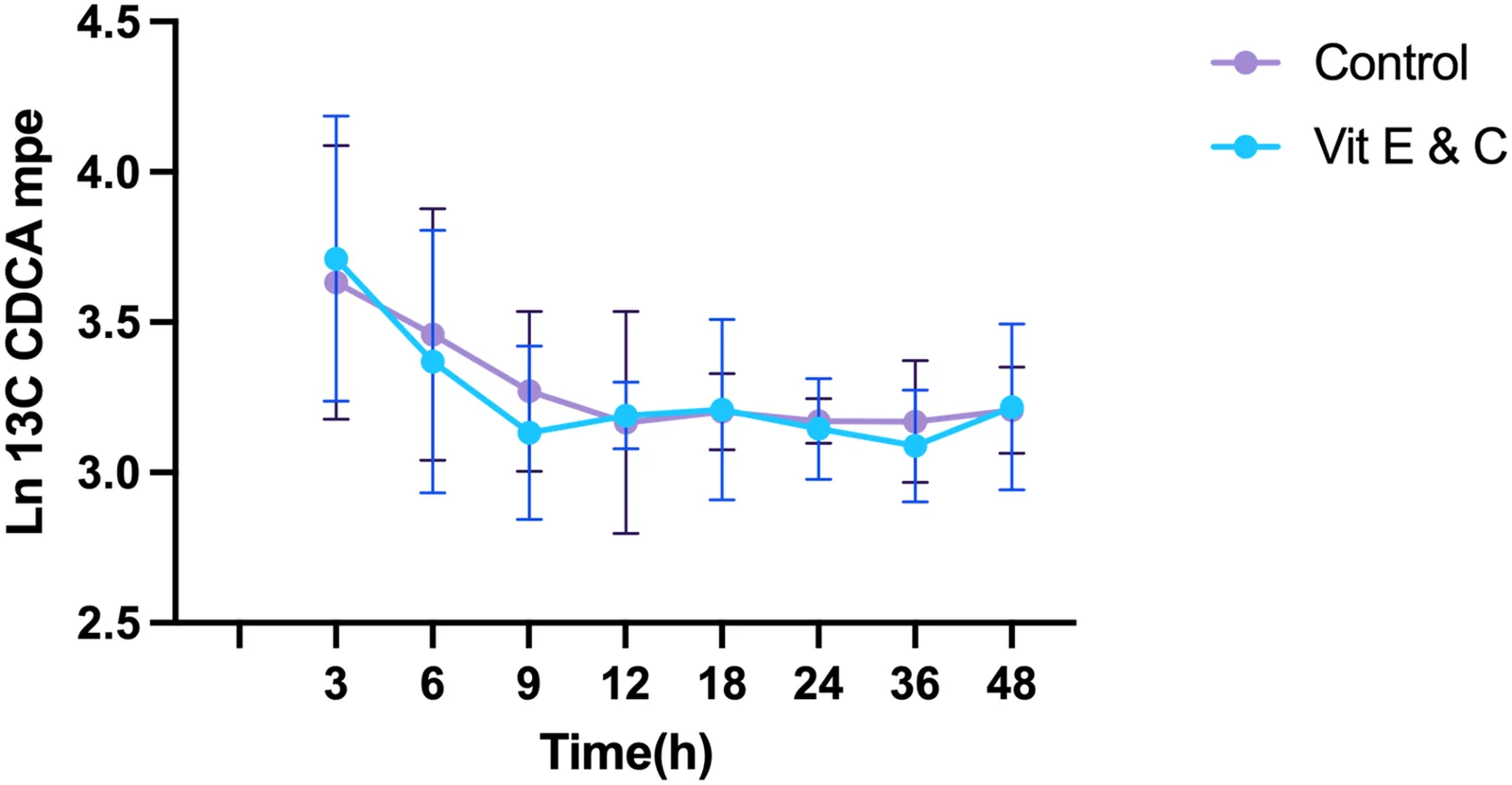
Kinetics of plasma ^13^C-CDCA clearance. Measurements were performed between days 8–10 in piglets receiving PN with or without vitamin E and C supplementation. Values are means ± SD; Control, n = 9; Vit E&C, n = 10. It shows time-dependent changes in ^13^C-CDCA enrichment expressed as natural log of mole percentage excess (Ln mpe) during the 48-hour period.

## Discussion

To our knowledge, this is the first *in vivo* study to evaluate combined vitamins E and C supplementation in a PN lipid emulsion using a neonatal piglet model. The present study demonstrated that vitamins E and C supplementation increased plasma and liver vitamin E levels and was associated with lower hepatic lipid accumulation and oxidative stress. These findings suggest enhanced hepatic antioxidant protection with co-supplementation, supporting the potential role of antioxidant-enriched PN formulations in mitigating PN-associated liver injury.

### The effect of vitamins E and C supplementation on liver parameters

A potential protective role for vitamins E and C was suggested by the lower hepatic TBARS in the Vit E&C group, indicating lower lipid peroxidation. Additionally, we found a trend toward higher antioxidant capacity with Vit E&C, suggesting that overall, these vitamins may mitigate oxidative stress.

Hepatomegaly is a well-documented characteristic of prolonged PN use, often associated with hepatic fat accumulation, inflammation and oxidative damage. Previous studies have consistently reported higher liver weights in PN-fed piglets compared to sow-reared counterparts [36–39]. While hepatic steatosis contributes to this liver enlargement, earlier findings also suggested a role of increased hepatic protein and DNA synthesis [38]. In our study, although overall liver weight only tended to be lower in the Vit E&C group, hepatic lipid content was lower in the Vit E&C group compared to the Control group, consistent with vitamin E’s known antioxidant role in reducing hepatic lipid accumulation and oxidative damage, as demonstrated in both experimental and clinical non-alcoholic fatty liver disease models [40,41]. Despite this reduction in hepatic fat, mean plasma and liver levels of TG and cholesterol were not significantly different between groups. This contrasts a previous piglet study that used Intralipid-based PN and found that vitamin E supplementation led to lower serum and hepatic TG and lower serum very low-density lipoprotein (VLDL) concentrations [6]. The discrepancy likely reflects differences in lipid emulsion composition; Intralipid (soybean oil based) can induce abnormally high TG and VLDL concentrations that were reduced by vitamin E, whereas SMOFlipid®, with its blend of soybean, olive, coconut, and fish oils, supports a more physiological lipid profile. Furthermore, SMOFlipid® contains anti-inflammatory omega-3 fatty acids, which may have contributed to improved hepatic lipid metabolism compared to Intralipid-based PN used in earlier studies. Although within-group regression analyses showed different patterns of association between hepatic lipid fractions and total liver fat, comparisons of the regression slopes did not support a treatment dependent difference in these relationships. Therefore, the mechanisms underlying the lower hepatic lipid accumulation observed with Vit E&C supplementation remain unclear. Importantly, lower hepatic lipid concentrations in Vit E&C piglets were evident after only 10 days of PN, a relatively short exposure compared with the two-week PN duration used in previous preterm piglet studies [36,37], suggesting that changes in hepatic lipid accumulation may occur earlier during PN than previously demonstrated. These findings suggest that antioxidant supplementation may exert early protective effects on hepatic lipid metabolism.

Despite marked differences in hepatic vitamin E content and lipid peroxidation, fractional liver protein synthesis did not differ between groups. The flooding dose method reflects incorporation of labeled phenylalanine into tissue specific proteins at a single time point, but underestimates rapidly secreted proteins, which account for 30–50% of liver proteins [42]. Previous work in rodent hepatocytes showed that oxidant stress promotes ApoB degradation and impairs VLDL secretion, while vitamin E supplementation prevents ApoB loss and restores lipoprotein export [43]. Thus, although we observed no difference between treatments in liver fractional protein synthesis rate, vitamin E and C supplementation may have enhanced hepatic secretion of ApoB-containing lipoproteins, a process our method would not detect. To test this possibility, future studies could employ stable isotope techniques to quantify VLDL-TG and VLDL-apoB-100 production in vivo [44], providing a more robust framework to examine treatment effects on secretory protein metabolism.

Bile acid clearance did not differ between groups, suggesting that hepatic dysfunction had not progressed to the point of compromising bile secretory function. This is consistent with the use of SMOFlipid®, a mixed-oil emulsion with low phytosterol content and relatively high α-tocopherol, which is associated with a lower risk of PN-related cholestasis in neonates [45–47].

These findings collectively suggest that vitamin E and C supplementation may mitigate hepatic lipid deposition primarily through localized antioxidant mechanisms rather than systemic lipid regulation, reinforcing their potential role in protecting against PN-associated liver dysfunction.

### Vitamin E distribution and storage by tissue

The liver is the primary site for nutrient storage and metabolism, while plasma reflects systemic nutrient availability. In our study, both plasma and liver showed significantly higher vitamin E concentrations in the supplemented group, indicating enhanced delivery and retention.

We included a group of sow-fed piglets as a physiological reference, representing the natural vitamin E status of term-born neonates not exposed to the oxidative stress of PN. Plasma vitamin E concentration in these piglets was similar to the range reported in studies of healthy domestic piglets [48,49]. At present, no empirically established plasma vitamin E targets exist for neonatal piglets [48]; thus, our sow-fed piglets provide the most appropriate baseline for evaluating vitamin E status in this model. Unlike healthy piglets, PN-fed neonates are exposed to greater oxidative stress, which may increase their requirement for dietary antioxidants supplied exogenously in PN. During the development of the PN-fed piglet model [22], Wykes et al. (1993) based their estimate of the intravenous vitamin E requirement on the NRC’s (1988) recommended oral intake of 1.2 mg/kg/d for 1–5 kg piglets. Their PN formulation delivered 135% of this estimate (1.5 mg/kg/day), which was assumed to be sufficient given no absorption coefficient was necessary with IV delivery; yet the adequacy of this concentration remains uncertain, as our study shows that PN piglets can receive even higher intakes of vitamin E but still fail to achieve consistently adequate plasma vitamin E levels. In our study, the control PN regimen delivered a total of 3.4 mg/kg/d (1.3 mg/kg/day from the PN multivitamin solution plus 2.1 mg/kg/day from SMOFlipid), or nearly triple the estimated requirement. Although PN control piglets exhibited mean plasma vitamin E concentrations that were almost two-fold higher than sow-fed piglets, the high variability within the PN group resulted in a third of the animals at or below the reference mean. Importantly, sow-fed piglets were not exposed to surgery or PN, and thus their values serve as a physiological reference rather than a direct comparator. Taken together, these findings suggest that standard PN delivery may not reliably ensure optimal vitamin E status. The considerable variability among PN-fed piglets also indicates that some individuals may not have achieved sufficient levels, potentially due to differences in oxidative stress, metabolic turnover or clearance rates. Notably, a previous study reported lower plasma vitamin E concentrations during PN compared to enteral feeding with both Intralipid and SMOFlipid [37]. In preterm piglets, plasma α‑tocopherol concentration was reduced by ~60% in a group supported with Intralipid-based PN compared to enterally-fed controls (1.88 ± 0.13 μg/mL vs. 4.61 ± 0.24 μg/mL, *P* < 0.05), with a significant reduction also observed in the SMOFlipid group (3.12 ± 0.39 µg/mL) [37]. Likewise, in adults on long-term PN, plasma and adipose tissue vitamin E concentrations remained suboptimal despite standard supplementation [50]. The oxidative demands during PN may exceed supply of vitamin E, raising concerns about the adequacy of current formulations in meeting antioxidant needs under stress conditions.

In our study, piglets receiving combined vitamin E & C supplementation achieved plasma vitamin E concentrations of 30 ± 12 μg/mL (vs 11 ± 5 μg/mL in Control), which were markedly higher than those reported in earlier PN studies using soy- or fish oil-based lipid emulsions without vitamin C. Across studies, plasma vitamin E concentrations in preterm piglets have ranged from ~3 μg/mL with SMOFlipid® and 2.7 μg/mL with Intralipid, increasing to ~7.6 μg/mL when Intralipid was supplemented with vitamin E, and ~4.6 μg/mL with an Intralipid-based PN at a dose similar to ours but without vitamin C [6,37,51]. By contrast, our values were 4–11 times higher than those reported in previous PN studies, despite comparable vitamin E dosing. These findings demonstrated that combined vitamin E and C supplementation effectively increased plasma vitamin E status during prolonged PN. However, differences in experimental conditions, PN formulations, sampling, and analytical methods across studies preclude attributing these differences specifically to vitamin C co-supplementation. The very high plasma concentrations exhibited by our co-supplemented group may have exceeded the ideal physiological range. While elevated plasma vitamin E could enhance antioxidant protection, the concentrations we observed approached the American Academy of Paediatrics’ upper intake limit for human infants. Whether such high values are beneficial or potentially excessive in piglets is unknown, as empirical reference ranges for adequacy and safety in PN-fed piglets have not been established. The markedly higher vitamin E levels in the co-supplemented group may partly reflect improved vitamin E stability in the PN solution prior to infusion, as vitamin C has been shown to reduce vitamin E degradation in vitro. Moreover, we have recently shown that peroxide formation is lower in PN solutions when supplemented with vitamin C and E [52]. In addition, vitamin C may have contributed to the preservation of vitamin E in vivo through mechanisms such as sparing or recycling of oxidized vitamin E. However, this cannot be confirmed with our study design. Future studies that isolate the individual contributions of vitamins E and C will be important to clarify mechanisms and guide safe and effective supplementation strategies.

Despite elevated vitamin E concentrations in plasma and liver, intestinal concentrations remained about 30-fold lower compared with the liver. This likely reflects the intravenous route of delivery bypassing the gut and favoring hepatic accumulation. No differences were measured between groups in small intestinal vitamin E content or lipid peroxidation, suggesting that antioxidant supplementation did not reduce gut oxidative stress.

### The effect of vitamins E & C supplementation on SMA blood flow and gut structure

This study also evaluated whether supplementing PN with vitamins E and C could mitigate the reduction in SMA blood flow that occurs with PN, preserving gut structure. However, blood flow was unchanged with vitamin supplementation, indicating no measurable effect of antioxidant treatment, and no difference in gut structure was found. As predicted, a progressive decline in blood flow was evident, with  ~ 35% reduction by day 3 and ~41% by day 4. This pattern is consistent with previous findings that splanchnic perfusion declines rapidly after PN initiation [2]. We hypothesized that enhanced antioxidant availability might preserve nitric oxide (NO) bioavailability and support vasodilation. In healthy endothelium, NO is produced via eNOS from L-arginine and oxygen and plays a crucial role in vascular tone regulation [53]. Under oxidative stress, however, eNOS can become uncoupled through S-glutathionylation, shifting production from NO to superoxide radicals; antioxidants have been suggested to mitigate eNOS uncoupling and support NO synthesis [54]. Despite this theoretical basis, our findings did not demonstrate a vasodilatory effect from antioxidant supplementation. It is possible that the dose or form of vitamin E and C used in this study was insufficient to counter oxidative stress or to maintain eNOS coupling. Additionally, cofactors such as tetrahydrobiopterin (BH4) are critical for eNOS function, and supplementation with BH_4_ in combination with antioxidants has been shown in other models to restore NO bioavailability and improve perfusion. For example, co-supplementation with BH_4_, L-arginine, and vit C significantly enhanced vascular recovery after hindlimb ischemia in rats by reducing oxidative stress, preserving NO bioavailability, and promoting collateral artery growth [55]. Although not studied in a PN setting, these findings suggest that combining antioxidants with eNOS cofactors or NO donors may be necessary to better sustain endothelial function and regional blood flow under prolonged PN. It is also possible that PN-induced reduction in blood flow is not mediated via eNOS. Future studies could also benefit from stable isotope techniques to measure NO production directly, such as the conversion of labeled L-arginine to NO and citrulline.

In the environment of reduced blood flow, the VH:CD ratio in both groups was approximately 2:1, below the normal physiological range of 3–5:1 [56], indicating notable villus shortening and blunting with PN feeding. These findings are consistent with previous studies demonstrating intestinal villus atrophy during PN [57]. While there were no effects of vitamins on villus structure, the Control data reinforce the well-established trend of progressive villus atrophy with prolonged PN feeding. The relatively small sample size may have limited the ability to detect modest treatment effects for some outcomes, and nonsignificant findings should therefore be interpreted cautiously.

Unlike the liver, where vitamin E accumulated and reduced lipid peroxidation, lipid peroxidation in the jejunum did not differ between groups. It may be that supplemented vitamins were unavailable to the gut, consistent with the bypass of enteral absorption during PN. This suggests that systemic antioxidant delivery during PN may not adequately reach intestinal tissues.

## Conclusion

As a clinically relevant translational model of neonatal parenteral nutrition, the neonatal piglet provides a valuable platform for evaluating nutritional interventions aimed at reducing PN-associated complications and informing the future development and clinical evaluation of antioxidant-enriched PN formulations. Vitamins E and C supplementation during prolonged PN increased plasma and hepatic vitamin E concentrations and was associated with lower hepatic oxidative stress, lipid peroxidation, and lipid accumulation in neonatal piglets. These findings support a potential protective role of antioxidant supplementation against early PN-associated hepatic injury, although additional biomarkers of liver injury may be needed to fully characterize hepatoprotective effects. Supplementation did not alter liver or intestinal protein synthesis, nor did it prevent SMA blood flow reduction or intestinal atrophy, suggesting limited impact on gut structure and function. Overall, these findings highlight the potential of antioxidant-enriched PN formulations to reduce hepatic oxidative stress, lipid peroxidation, and lipid accumulation during PN, while also underscoring the need for additional strategies to support gut structure and function during PN.

## Supporting information

S1 Fig Association between initial body weight and hepatic FRAP. Relationship between initial body weight and hepatic ferric reducing antioxidant power (FRAP) in neonatal piglets. Simple linear regression was performed using pooled data from both treatment groups (n = 20; R^2^ = 0.06, P = 0.31).
(TIFF)

S1 File Supplementary Methods. Supplementary methods for the analysis of liver phosphatidylcholine, bile phosphatidylcholine, total bile acids in bile, and bile cholesterol.
(DOCX)

S1 Table Composition of the elemental diet. (DOCX)

S2 Table Bile cholesterol, bile acids, phosphatidylcholine and liver phosphatidylcholine. (DOCX)
