# Supplementary material for: Vitamins E and C supplementation reduces hepatic lipid peroxidation and steatosis in neonatal Yucatan piglets receiving prolonged parenteral nutrition
Source: PLoS One. 2026 Nov 2;21(11):e0361191. doi: 10.1371/journal.pone.0361191 (PMC13726914; doi:10.1371/journal.pone.0361191)
Supplement: Supplementary file 1 [file pone.0361191.s002.docx]

# **S1 File. Supplementary Methods.**

## **Liver phosphatidylcholine.**

## Liver lipid extracts were analyzed for phosphatidylcholine (PC) content using thin-layer chromatography (TLC) followed by a modified Bartlett phosphate assay (Bartlett, 1959; Stadler et al., 2013). Aliquots (10 µL) were applied to Silica Gel-G60 TLC plates, developed in a chloroform: methanol: acetic acid: water solvent system (25:15:4:2, v/v/v/v), and visualized in an iodine chamber. Bands corresponding to PC and phosphatidylethanolamine (PE) were scraped, digested in 70% perchloric acid at 180 °C for 2 h, and quantified calorimetrically using the Bartlett method with phosphate standards prepared from potassium phosphate monobasic. Absorbance was measured at 815 nm.

**Bile phosphatidylcholine.**

PC concentrations in bile were determined with a commercial colorimetric kit (Phosphatidylcholine Assay Kit, ab83377; Abcam, Cambridge, UK) according to the manufacturer’s instructions. Samples (1 µL) were assayed in duplicate, and absorbance was measured at 570 nm. Concentrations were calculated against a standard curve prepared from supplied phospholipid standards.

## **Total bile acids in bile.**

Total bile acid concentrations were measured using a Total Bile Acids (TBA) Assay Kit (Sigma-Aldrich, St. Louis, MO, USA). Bile samples (20 µL) were analyzed in duplicate with sodium cholate calibration standards. The enzymatic reaction was detected fluorometrically at λ_ex 530 nm/λ_em 585 nm, and bile acid concentrations were derived from the standard curve.

## **Bile cholesterol.**

Cholesterol concentrations were determined using an enzymatic colorimetric assay kit (Sekisui Diagnostics, Charlottetown, PEI, Canada). Bile samples were diluted 1:2 in distilled water before analysis. Samples and standards (prepared from a 100 mg/dL cholesterol stock) were assayed in duplicate, and absorbance was measured at 505 nm. Concentrations were calculated against the standard curve.
