# Supplementary material for: Vitamins E and C supplementation reduces hepatic lipid peroxidation and steatosis in neonatal Yucatan piglets receiving prolonged parenteral nutrition
Source: PLoS One. 2026 Nov 2;21(11):e0361191. doi: 10.1371/journal.pone.0361191 (PMC13726914; doi:10.1371/journal.pone.0361191)
Supplement: Supplementary file 2 [file pone.0361191.s003.docx]

## **S1 Table. Composition of the elemental diet**.

| **composition of elemental diets** | g/l |
| --- | --- |
| L-Amino acids | 54.90 |
| D-Glucose | 90.30 |
| Trihydrate K2HPO | 1.57 |
| Monobasic KH2PO4 | 1.09 |
| Potassium acetate | 1.47 |
| NaCl | 2.17 |
| MgSO4 | 0.78 |
| ZnSO4 | 0.09 |
| Calcium gluconate | 6.41 |
|  |  |
| **Composition of admixtures** |  |
| Vitamins | dose /kg body weight/d |
| Ascorbic acid, mg | 15.33 |
| Vitamin A, µg | 134.10 |
| Vitamin D, µg | 1.92 |
| Thiamine (as hydrochloride), mg | 0.26 |
| Riboflavin (as phosphate), mg | 0.27 |
| Pyridoxine hydrochloride, mg | 0.19 |
| Niacinamide, mg | 3.70 |
| d-Panthenol, mg | 1.10 |
| Vitamin E (alpha tocopherol), mg | 1.34 |
| Vitamin K1, mg | 0.04 |
| Biotin, µg | 38.31 |
| Folic Acid, µg | 26.82 |
| Vitamin B12, µg | 0.19 |
|  |  |
| Trace Element Mix | mg/ kg body weight/d |
| Zinc (as ZnSO4.7H2O) | 38.97 |
| Copper (as CuSO4.5H2O) | 2.99 |
| Manganese (as MnSO4.H2O) | 1.78 |
| Chromium (as CrCl3.6H2O) | 0.05 |
| Selenium (as SeO2) | 0.05 |
| Iodide (as NaI) | 0.02 |
|  |  |
| Iron Dextran | 3.00 |
