# Supplementary material for: Vitamins E and C supplementation reduces hepatic lipid peroxidation and steatosis in neonatal Yucatan piglets receiving prolonged parenteral nutrition
Source: PLoS One. 2026 Nov 2;21(11):e0361191. doi: 10.1371/journal.pone.0361191 (PMC13726914; doi:10.1371/journal.pone.0361191)
Supplement: Supplementary file 3 [file pone.0361191.s004.docx]

## **S2 Table: Bile cholesterol, bile acids, phosphatidylcholine and liver phosphatidylcholine.**

| Parameter | Control | Vit E & C | P value |
| --- | --- | --- | --- |
| Bile - cholesterol (mmol/L) | 4.22 ± 2.26 | 5.91 ± 2.77 | 0.21 |
| Bile - bile acids (mmol/L) | 0.47 ± 0.64 | 0.23 ± 0.18 | 0.45 |
| Bile - phosphatidylcholine (mmol/L) | 10.62 ± 5.37 | 12.18 ± 3.69 | 0.56 |
| Liver - phosphatidylcholine (µmol/g) | 6.52 ± 5.15 | 9.12 ± 5.90 | 0.33 |

Values are means ± SD; For liver phosphatidylcholine, n = 10 per group; for bile, n = 9 (control) and n = 6 (vitamin-supplemented) because sufficient bile could not be collected from all piglets. P values were determined using unpaired t-tests.
