# Supplementary material for: Vitamins E and C supplementation reduces hepatic lipid peroxidation and steatosis in neonatal Yucatan piglets receiving prolonged parenteral nutrition
Source: PLoS One. 2026 Nov 2;21(11):e0361191. doi: 10.1371/journal.pone.0361191 (PMC13726914; doi:10.1371/journal.pone.0361191)
Supplement: Supplementary file 4 [file pone.0361191.s005.docx]

**Response to the Editor and Reviewers**

**Manuscript title:** *Supplementing SMOFlipid® with Vitamins E and C Reduces Liver Damage in Neonatal Piglets during Prolonged Parenteral Feeding*

Dear Editor,

We sincerely thank you and the reviewers for the careful evaluation of our manuscript and for the thoughtful and constructive comments. We appreciate the time and effort invested in reviewing our work. We have carefully revised the manuscript in response to all comments. Our point-by-point responses are provided below, and all manuscript changes are indicated in the revised manuscript.

Journal requirements from PLOS ONE

1. Please ensure that your manuscript meets PLOS ONE's style requirements, including those for file naming.
2. When completing the data availability statement of the submission form, you indicated that you will make your data available on acceptance. We strongly recommend all authors decide on a data sharing plan before acceptance, as the process can be lengthy and hold up publication timelines. Please note that, though access restrictions are acceptable now, your entire data will need to be made freely accessible if your manuscript is accepted for publication. This policy applies to all data except where public deposition would breach compliance with the protocol approved by your research ethics board. If you are unable to adhere to our open data policy, please kindly revise your statement to explain your reasoning and we will seek the editor's input on an exemption. Please be assured that, once you have provided your new statement, the assessment of your exemption will not hold up the peer review process.
3. We note that you have included the phrase “data not shown” in your manuscript. Unfortunately, this does not meet our data sharing requirements. PLOS does not permit references to inaccessible data. We require that authors provide all relevant data within the paper, Supporting Information files, or in an acceptable, public repository. Please add a citation to support this phrase or upload the data that corresponds with these findings to a stable repository (such as Fig share or Dryad) and provide and URLs, DOIs, or accession numbers that may be used to access these data. Or, if the data are not a core part of the research being presented in your study, we ask that you remove the phrase that refers to these data.
4. We note that you have referenced (i.e. White JMB. [26]) which has currently not yet been accepted for publication. Please remove this from your References and amend this to state in the body of your manuscript: (ie “Bewick et al. [Unpublished]”) as detailed online in our guide for authors.
5. Please include captions for your Supporting Information files at the end of your manuscript, and update any in-text citations to match accordingly. Please see our Supporting Information guidelines for more information
6. If the reviewer comments include a recommendation to cite specific previously published works, please review and evaluate these publications to determine whether they are relevant and should be cited. There is no requirement to cite these works unless the editor has indicated otherwise.

**Responses to journal requirements**

1. We thank the journal for this reminder. We have reviewed the manuscript and ensured that it meets the PLOS ONE style requirements throughout, including the requirements for file naming.
2. We will ensure that the data underlying the findings reported in the manuscript are made publicly available in accordance with PLOS ONE’s Data Availability Policy upon acceptance.
3. We thank the journal for this comment. The phrase “data not shown” has been removed, and the liver dry matter data have now been included in Table 1, and the regression analysis between initial body weight and hepatic FRAP has been provided as S1 Fig.
4. We thank the journal for this clarification. The unpublished reference (White JMB) has been removed from the reference list. This reference was previously cited in two places in the manuscript. First, it was cited in relation to the methodology used for the TBARS analysis; this section has been expanded to provide the methodological details directly, and the citation to the unpublished work has therefore been removed. Second, the reference was used to support a quantitative comparison of intestinal villus morphology. In response to Reviewer 2’s concern regarding the use of unpublished data for this quantitative comparison, the comparison and its associated citation have been removed. Consequently, the revised manuscript no longer contains statements relying on White JMB [Unpublished], and no unpublished citation remains in the body of the manuscript.
5. We thank the journal for this clarification. Captions for all Supporting Information files (S1 Fig, S1 File, S1 Table, and S2 Table) have been added to the manuscript, and the corresponding in-text citations have been checked for consistency.
6. Noted. No specific additional published works were recommended for citation by the reviewers.

**Reviewer 1**

**Reviewer1- comment 1- Abstract**

**Reviewer requests:** In the abstract, it would be important to clearly indicate that neonatal piglets were used as an animal model to study complications of parenteral nutrition in neonates. This information is essential for the reader at the abstract stage.

Additionally, the phrase “we hypothesized” could be replaced with a more neutral statement of the study aim. In many experimental abstracts, a clear presentation of the study objective improves clarity and formal tone.

**Response-** We thank the reviewer for these helpful suggestions. The Abstract has been revised to clearly indicate that neonatal Yucatan miniature piglets were used as an established animal model for investigating PN-associated complications in neonates. In addition, the hypothesis statement has been replaced with a more neutral statement describing the objective of the study, as suggested.

**Reviewer1 - Comment 2 – Introduction**

**Reviewer requests -** The authors should explicitly state that the study was conducted on an animal model (neonatal piglets), which serves to investigate parenteral nutrition complications in neonates. This information should be presented clearly at the beginning of the introduction so that the reader immediately understands the research context.

The hypothesis in the introduction is not clearly formulated. It should define the expected impact of vitamin E and C supplementation on reducing oxidative stress and liver and intestinal injury during parenteral nutrition.

**Response -** The Introduction has been revised to clearly state, near the beginning of the manuscript, that neonatal Yucatan miniature piglets were used as an established translational animal model for investigating PN-associated complications in neonates.

In addition, the study hypothesis has been revised to more clearly define the expected effects of vitamin E and C supplementation on oxidative stress, liver injury, intestinal blood flow, and gut atrophy during prolonged PN.

**Reviewer 1- Comment 3 – Materials and Methods**

**Reviewer Requests -** The starting body weight of the neonatal piglets should be clearly reported.

In the section describing the analyses, the authors should briefly describe the procedure of each method rather than referring only to previous publications (e.g., refs.19-25).

**Response** The initial body weight of the piglets (grouped by treatment) was reported in Table 1. There was no difference in initial weight by treatment, so we have added the pooled body weight (and SD) at entry into the study. This can be found in the **"Piglets, surgical procedures and daily care"** section of the Materials and Methods section.

In addition, we have expanded the descriptions of the analytical and experimental procedures throughout the Materials and Methods to reduce reliance on citations to previous publications. Specifically, we have added methodological details describing the surgical catheterization and SMA flow-probe implantation procedures, PN diet formulation and preparation, histological processing and morphometric assessment, and the FRAP and TBARS assays, while retaining citations to the original publications where appropriate for additional methodological detail.

**Reviewer 1- Comment 3 - Results**

**Reviewer Requests -** The title of Table 4 does not reflect the data presented and requires correction. Values should be presented consistently throughout tables and text, for example: 0.001; 0.010; 1.01; 11.11; 100.1; 1000.1. These comments also apply to p-values, there is no need to report, for instance, p = 0.0015; rounding to 0.002 is sufficient.

**Response –** The title of Table 4 has been revised to accurately reflect the data presented. Thank you for pointing out this error.

Numerical values throughout the manuscript, including those presented in the text and tables, have been reviewed for consistency and appropriate rounding. P-values have also been reviewed to avoid reporting unnecessary decimal precision.

**Reviewer 1- Comment 4 - Discussion**

**Reviewer Requests -** It is unclear why the authors placed a summary of the results at the beginning of the discussion. I suggest that, after corrections, this section should serve as a formal summary of the findings rather than an introduction to the discussion.

**Response –** We thank the reviewer for this helpful suggestion. The opening paragraph of the Discussion has been revised to provide a more concise summary of the principal findings and a clearer transition into the discussion. The remainder of the Discussion focuses on interpreting these findings in the context of previous literature.

**Reviewer 1- Comment 5 - Conclusion**

**Reviewer Requests -** The current conclusion is too general. The hepatoprotective effect depends on multiple factors, including reduction of hepatocyte injury, limitation of fat accumulation in the liver, reduction of oxidative stress, improvement of liver metabolic function, and lowering of liver injury markers: ALT, AST, ALP, GGT, LDH, BIL. Therefore, I believe that the authors, having access to biological material (plasma), could consider performing additional analyses of these markers, which would certainly increase the value of the study and strengthen the conclusions regarding hepatoprotective effects.

Moreover, as previously mentioned, the manuscript lacks a clear emphasis on the role of piglets as an animal model. The current results could be discussed more broadly in the context of translational relevance, e.g., relating to human health, clinical applications, or comparison with other liver models. Highlighting this aspect would increase the applied significance of the study and help the reader understand the purpose of the experiment.

**Response –**We agree that additional biochemical markers of liver injury could provide a more comprehensive assessment of hepatoprotective effects. Unfortunately, insufficient plasma remains from this study to perform additional analyses, and funding is not available for further assays. We have therefore revised the Conclusion to focus on the hepatic outcomes measured in this study and acknowledge that additional biomarkers are needed to more comprehensively characterize hepatoprotective effects.

We have also revised the Conclusion to emphasize the translational relevance of the neonatal piglet model for studying PN-associated complications and evaluating nutritional interventions.**Reviewer 2**

**Reviewer 2- Comment 1**

**Reviewer Requests -** The "synergy" framing is not supported by the design. The Introduction and aims describe investigating the synergistic effects of E and C, but the study has only two arms (combined vs. control) — no E-only or C-only arm. Synergy cannot be established without those arms. The authors acknowledge this, but they then infer synergy largely by comparing their plasma vitamin E values to historical literature ("7–10 times higher... despite comparable dosing"). Cross-study comparisons are confounded by assay, animal, timing, and formulation differences and cannot substitute for a factorial design. Please reframe throughout: the study demonstrates that co-supplementation is hepatoprotective, not that E and C act synergistically. The title (which avoids "synergy") is appropriate; the aims and parts of the Discussion overreach.

**Response –** We thank the reviewer for this important comment and agree that the present study design does not allow synergistic effects of vitamins E and C to be established. Accordingly, we have revised the manuscript throughout to remove statements implying synergy and have reframed the Introduction and Discussion to focus on **combined vitamin E and C co-supplementation**. We have also removed the inference that the higher plasma vitamin E concentrations observed relative to previous studies support a synergistic effect, as cross-study differences cannot establish such an interaction. Potential interactions between vitamins E and C are now discussed only as possible mechanisms, and we clarify that the present design cannot determine their individual or interactive effects.

**Reviewer 2- Comment 2**

**Reviewer Requests –**

Multiple comparisons without correction.

Numerous t-tests are run across Tables 1–4 (growth, organ weights, morphology, lipids, peroxidation, vitamin E).

The two significant hepatic outcomes are P=0.02 (total lipid) and P=0.03(MDA); neither would survive a family-wise correction across the panel.

Please state the analysis plan explicitly (which comparisons were pre-specified vs. exploratory) and either apply an appropriate correction or clearly designate the exploratory outcomes and soften the corresponding claims.

**Response- We thank the reviewer for raising this important statistical consideration. The outcomes reported in Tables 1–4 were pre-specified as part of the experimental protocol and were not selected following examination of the treatment-group results. The growth and organ/tissue measurements reported in Table 1 were collected according to the planned study procedures at baseline and/or necropsy on day 10. Similarly, the intestinal measurements reported in Table 2 were obtained at necropsy, with jejunal samples collected and fixed for subsequent histological assessment of villus height and crypt depth. The hepatic and plasma lipid and oxidative stress outcomes reported in Table 3 and vitamin E status reported in Table 4 were also pre-specified analytical outcomes of the study. In particular, hepatic total lipid and TBARS, highlighted by the reviewer, were included as measures of hepatic lipid accumulation and oxidative stress, respectively.**

Nevertheless, we recognize the concern regarding multiple outcome testing and have revised the Abstract, Discussion, and Conclusion to provide a more cautious interpretation of these findings.

**Reviewer 2- comment 3**

**Reviewer Requests –** Invalid inference from the regression comparison (Fig 2). The claim of a "shift away from cholesterol-driven lipid deposition" rests on observing that cholesterol is a significant predictor in Controls (R²=0.47, P=0.04) but not in Vit E&C (R²=0.08, P=0.4), and the reverse for TG. Comparing whether each within-group regression reaches significance is not a valid test of a between-group difference. To support this claim, you must formally test the group × predictor interaction (i.e., whether slopes/intercepts differ between groups) in a single model. With n=10 per group and modest R² values, this interpretation should be presented cautiously and, in the abstract, the "shift away from cholesterol driven lipid deposition “statement should be softened or removed unless the interaction test is significant.

**Response -** We thank the reviewer for identifying this issue. We agree that differences in statistical significance between the within-group regressions do not establish a difference in the relationships between treatment groups. Regression slopes and intercepts were formally compared between the Control and Vit E&C groups. The slopes did not differ significantly between treatment groups for either hepatic cholesterol (P = 0.33) or triglycerides (P = 0.94). Accordingly, we have removed the interpretation that antioxidant supplementation resulted in a “shift away from cholesterol-driven lipid deposition” from the Abstract and Discussion. The Results and Statistical Analysis sections have also been revised to report the formal comparison of the regression lines and to distinguish the within-group associations from the between-group comparisons.

**Reviewer 2- comment 4**

**Reviewer Requests –**

i. Sample-size accounting and varying n. Twenty-one enrolled, 20 completed, one euthanized on day 6 — but both analysis groups are reported as n=10 (total 20). Which group did the excluded animal belong to, and how were exclusions handled?
ii. The n also varies by outcome without explanation: blood flow n=8/group (Fig 1), bile acid n=7/group (Fig 4), plasma vitamin E n=9–10 (Fig 3).
iii. Please provide a clear participant-flow account (an ARRIVE-style flow diagram or a short paragraph) stating allocation, dropouts, probe/assay failures, and inclusion/exclusion criteria.
iv. No a prior power/sample-size justification is given; given the many null results, a brief statement on power (or an acknowledgment that null results may be underpowered) is needed.

**Response –**

i. We thank the reviewer for requesting clarification. One piglet allocated to the Vit E&C group was humanely euthanized on study day 6 due to clinical deterioration and was excluded from all analyses. An additional piglet was subsequently enrolled to maintain the planned sample size. Thus, 21 piglets entered the study, with 20 completing the study (Control, n = 10; Vit E&C, n = 10). The animal allocation and study completion details have now been clarified in the Materials and Methods.
ii. SMA blood flow analyses included eight piglets per treatment group. Four animals (two per treatment group) were excluded from the longitudinal analysis because of absent or incomplete recordings resulting from technical loss of the implanted flow-probe signal. Because the probes were surgically implanted around the SMA, loss of probe signal during the study could not be corrected by replacing the probe, resulting in a lower sample size for this analysis.

Upon rechecking the bile acid dataset, we identified an error in the originally reported sample size. The 13C-CDCA clearance analysis included 9 Control and 10 Vit E&C piglets, rather than 7 piglets per group. One Control piglet did not undergo the clearance study because the femoral catheter was nonfunctional at the time of the procedure. The jugular catheter was reserved for PN infusion and subsequent study procedures and therefore was not used for bolus administration and serial blood sampling. The figure legend has been corrected accordingly.

For plasma vitamin E, re-evaluation of the dataset confirmed that data were available for all 20 piglets. The analysis has therefore been updated to include **10 piglets per treatment group**, and the corresponding figure and legend have been revised accordingly.

The sample sizes and reasons for missing data have now been clarified in the relevant sections of the manuscript and figure legends.

iii. We thank the reviewer for this suggestion. We have added a participant-flow description to the manuscript to clarify study completion and outcome-specific sample sizes. The Ethics Statement accounts for the 21 piglets that entered the study and the 20 that completed it (Control, n = 10; Vit E&C, n = 10). The Statistical Analysis section has been revised to describe outcome-specific missing measurements due to technical or procedural limitations, including SMA blood flow (n = 8/group) and ^13^C-CDCA clearance (Control, n = 9; Vit E&C, n = 10). Outcome-specific sample sizes are also reported in the corresponding figure legends.
iv. We thank the reviewer for this comment. The sample size was determined a priori based on previous variance estimates for SMA blood flow in neonatal piglets, using a significance level of 0.05 and 80% power to detect a 15% difference in SMA blood flow. The Statistical Analysis section has been revised to include this sample-size justification.

**Reviewer 2- comment 5**

**Reviewer Requests –** Randomization and blinding are underreported. Histology was blinded, which is good, but how animals were allocated to Control vs. Vit E&C is not stated, and blinding of biochemical assays is not described. Please report the randomization method and blinding for all outcome assessments. Aligning the manuscript with the ARRIVE 2.0 checklist would resolve items 4 and 5 together.

**Response –** Randomization of piglets into the two treatment groups was blocked by littermate pairs. Investigators were aware of treatment allocation during the conduct of the experiment because treatment-specific PN formulations were administered to each piglet. Biological samples were identified by a study code rather than treatment group, and personnel performing the assays were unaware of treatment allocation. Histological assessments were performed blinded to treatment allocation. The Materials and Methods have been revised to clarify these procedures.

The manuscript was also reviewed against the ARRIVE 2.0 Essential 10 checklist, and relevant sections were revised to improve reporting of animal allocation, exclusions, sample sizes, and blinding procedures.

**Reviewer 2- comment 6**

Minor issues (factual/reporting corrections)

1. Abstract P-value error. The abstract states hepatic lipid accumulation was reduced at "P<0.01," but Table 3 reports P=0.02. It should read P<0.05. Please reconcile.-
2. Table 4 units are wrong. Vitamin E is listed as "(g/g)" for liver and jejunal mucosa; values of 120–203 can only be μg/g.Correct throughout.
3. Vitamin E dose inconsistency. Methods state control vitamin E = 3.5 mg/kg/d; the Discussion states 3.4 mg/kg/d (1.3 +2.1). Make consistent and show the arithmetic once.
4. Reference 26 is cited as unpublished thesis data yet used for a quantitative morphology comparison. Given PLOS's verifiability standards, consider whether this can be supported by a published source or the data made available.
5. Fig 1 legend presents group-specific lines but pooled statistics ("pooled mean line not shown"). This is confusing; clarify what the asterisks mark and consider plotting the pooled line or clearly labeling the two-stage analysis.
6. Typos: "Jejuanl" (Table 2 → Jejunal); "exanguination" (ethics statement → exsanguination, correct elsewhere); check "two- three weeks" hyphenation.
7. A light copyedit pass is warranted, though the English is otherwise clear and publication-standard.

**Response –**

1. Corrected. The abstract now reports **P < 0.05**, consistent with the **P = 0.02** reported in Table 3.
2. We verified the units reported in Table 4 and confirm that vitamin E concentrations in liver and jejunal mucosa are expressed as **μg/g** in the original submission. No correction to the units in Table 4 was therefore required. We also searched the document to see if “g/g” was used elsewhere in error, and found nothing.
3. Corrected. The total vitamin E intake in the Control group was **3.4 mg/kg/d (1.3 mg/kg/d from the PN multivitamin preparation + 2.1 mg/kg/d from SMOFlipid®).** The Methods and Discussion have been revised for consistency.
4. We thank the reviewer for this comment. We agree that the quantitative comparison should be supported by a verifiable source. We have therefore removed the quantitative comparison based on unpublished data and revised this section to describe the findings in relation to published studies of intestinal villus atrophy during PN.
5. We thank the reviewer for pointing out that the presentation in Fig. 1 was unclear. The two treatment groups are displayed separately in Fig. 1, whereas the follow-up Dunnett’s analysis was performed on the pooled data after a significant main effect of time was detected. We have revised the figure by removing the comparison brackets and placing the significance symbols above the corresponding study days. The figure legend has also been revised to clarify that these asterisks indicate significant differences from baseline (day 0) in the pooled analysis.
6. We thank the reviewer for identifying these typographical and reporting issues. The indicated terms and hyphenation have been checked and corrected where necessary.
7. The manuscript has also undergone a final copyedit to correct minor grammatical, typographical, and formatting inconsistencies.
